# The Effect of Human Growth Hormone on Endometrial Growth in Controlled Ovarian Hyperstimulation Cycles

**DOI:** 10.3390/jpm12121991

**Published:** 2022-12-01

**Authors:** Brindha Bavan, Rebecca M. Gardner, Wendy Y. Zhang, Lusine Aghajanova

**Affiliations:** 1 Department of Obstetrics & Gynecology, Division of Reproductive Endocrinology & Infertility, Stanford University, Sunnyvale, CA 94087, USA; 2 Quantitative Sciences Unit, Stanford University School of Medicine, Palo Alto, CA 94304, USA

**Keywords:** human growth hormone, endometrial lining, endometrial thickness, endometrial growth, infertility

## Abstract

This study aims to compare endometrial growth before and after the addition of human growth hormone (hGH) in controlled ovarian hyperstimulation (COH) cycles. A 5-year retrospective cohort study of patients treated with hGH to improve oocyte development during COH cycles was conducted. Each patient’s cycle without hGH immediately preceding cycle(s) with hGH was used for patients to serve as their own controls. Primary outcome was absolute growth in endometrial thickness from pre-stimulation start to day of hCG trigger. Mixed-model regression analysis controlled for patient correlation over repeat cycles and potential confounders. 80 patients were included. Mean age was 39.7 years; mean BMI was 23.8 kg/m^2^. Majority of patients were nulliparous, non-smoking, and White or Asian. Most common diagnosis was diminished ovarian reserve. Endometrial growth was compared between 159 COH cycles with hGH and 80 COH control cycles; mean increase was 4.5 mm and 3.9 mm, respectively-an unadjusted difference of 0.6 mm (95% CI: 0.2–1.1, *p* = 0.01). After adjusting for demographic/clinical factors, hGH was associated with 0.9 mm greater endometrial growth (0.4–1.4, *p* < 0.01). Absolute increase in endometrial thickness was higher in COH cycles that included hGH. Further prospective studies in embryo transfer cycles are needed.

## 1. Introduction

The success of assisted reproductive technologies depends on multiple factors: the availability and quality of embryos, adequate development of the endometrium, and synchronization of the two to ensure endometrial receptivity to embryo implantation [1]. While significant progress has been made with regard to embryology, management of endometrial lining and the uterine aspect of implantation remains challenging in many cases [2].

Various factors-hormonal, vascular, immune-have an impact on endometrial thickness [3]. The adequacy of endometrial lining is clinically defined by its thickness and pattern as determined by transvaginal ultrasonography, with sagittal measurement > 7 mm and triple-line pattern correlating with improved implantation and pregnancy outcomes [4,5,6]. Prior evidence has supported that increased growth in endometrial lining during IVF cycles from thinnest prior to gonadotropic start and thickest at time of hCG trigger is a positive prognostic indicator of pregnancy outcomes as well [5].

Researchers have investigated the impact of human growth hormone (hGH) on fertility for over three decades now, but its benefit and role remain a source of ongoing debate [7,8,9,10,11]. The diverse utilization of hGH in fertility treatments originally focused on improving oocyte quality, but now encompasses emerging data showing positive effects on the endometrial environment and its receptivity. Studies have confirmed hGH and its receptor expression in the glandular epithelial cells of the endometrium, suggesting a key role in its functions such as growth, vascularity, and implantation [6,12]. Investigators are now exploring hGH as a potential treatment for conditions such as thin endometrial lining or recurrent implantation failure [13,14,15]. However, the current available evidence is mostly translational with limited clinical data exploring the effect of hGH on uterine lining development in patients [16].

To address this specific gap in knowledge, our study aims to compare endometrial growth during controlled ovarian hyperstimulation (COH) cycles in poor-responder patients before and after the addition of hGH. To our knowledge, this study design is the first of its kind to focus on the endometrial impact of hGH supplementation in individuals who have undergone multiple COH cycles with and without such treatment, effectively allowing patients to serve as their own controls. Our goal at the outset was to further elucidate whether hGH increases endometrial thickness. We found that COH cycles with hGH had a greater absolute endometrial growth compared to those without hGH. Further prospective studies are warranted to consider hGH as an adjunct therapy for endometrial preparation during embryo transfer cycles in eligible patients.

## 2. Materials and Methods

### 2.1. Retrospective Cohort Study Design

We identified patients who underwent COH protocols that included hGH between July 2014 and June 2019 at a single academic fertility center (Institutional Review Board-approved protocol #52753). It is not our clinic’s usual practice to prescribe hGH during a patient’s first COH cycle. Typically, a patient will demonstrate poor oocyte yield, fertilization rate, and/or blastocyst conversion during an initial cycle before hGH is considered as an adjunct therapy in a subsequent cycle. Therefore, each subsequent COH cycle that included hGH was included in this study to comprise the intervention arm, and the most recent preceding COH cycle that did not include hGH was included in this study to comprise the control arm. This approach allowed for patients to serve as their own controls with the shortest time gap between compared cycles.

The patients underwent conventional ovarian stimulation cycles using standard protocols per the primary physician. To administer hGH, patients self-injected Omnitrope^®^ (Novartis, East Hanover, NJ, USA) 8 IU daily into the subcutaneous tissue at the same time as gonadotropin stimulation medications, between 8:00–10:00 PM. Patients stopped hGH after 10 days or on the day of trigger injection, whichever occurred sooner. Baseline GH or IGF-1 levels were not routinely checked before treatment, as this is not part of clinical practice.

Demographic factors including age at retrieval (<35, 35–37, 38–40, and >40 years old), BMI (kg/m^2^), race (White, East Asian, South Asian, Black or African American), ethnicity (Hispanic/Latino, non-Hispanic/Latino) and smoking status were collected. Reproductive health characteristics were also studied including obstetrical history, Society of Assisted Reproductive Technology (SART) diagnosis, anti-Mullerian hormone (AMH) serum level (ng/dL), and antral follicle count collected within 1 year of COH cycle start. Finally, cycle specific details including COH protocol (antagonist, microdose flare, clomid antagonist, letrozole antagonist, and long Lupron), baseline antral follicle count, total gonadotropin dose, day of trigger/peak estradiol serum level (pg/mL), and trigger injection type/dose were documented as well.

The primary outcome of this study was endometrial growth, specifically the absolute change in endometrial thickness assessed by transvaginal ultrasound from the thinnest reading (at baseline or first scan after stimulation start) to the thickest reading (typically on the day of trigger/peak estradiol level) in millimeters. 

### 2.2. Statistical Analyses

We calculated descriptive statistics at the patient-level-means, standard deviations, medians and interquartile ranges (IQR) are reported for continuous variables while frequencies and percentages are reported for categorical variables. We calculated means and standard deviations for endometrial growth for each category of age at retrieval, race, diminished ovarian reserve (DOR) diagnosis, and COH protocol. We calculated absolute standardized differences (ASD) to determine if there were meaningful differences in endometrial growth between groups. Values of 0.2, 0.5, and 0.8 correspond to small, moderate, and large differences, respectively [17].

In order to account for the correlation between cycles per patient, we implemented linear mixed modeling to assess differences in change in endometrial lining between control and hGH cycles. Both unadjusted and adjusted (demographics, reproductive health history, and cycle specific details including age, BMI, DOR status, specific COH protocol, total gonadotropin dose, and day of trigger/peak estradiol level) were completed. We calculated point estimates for each independent variable, 95% confidence intervals (CIs), and *p*-values. All analyses were conducted using R version 3.6.2 including the *lme4* and *lmerTest* packages for linear mixed modeling [18,19,20]. All statistical tests were two-sided and performed at the 0.05 significance level.

## 3. Results

A total of 80 patients met study inclusion criteria. The majority of patients were of advanced maternal age (mean 39.7 years) with diminished ovarian reserve (69%), White or Asian (98%), nulliparous (75%), non-obese (mean BMI 23.8), and non-smoking (99%). Median age for non-hGH COH cycle group was 39.2 years (IQR = 37, 42) and median age for hGH COH cycle group was 39.9 years (IQR = 38, 42.5). The full breakdown of demographic and cycle characteristics is included in Table 1.

Mean endometrial thickness at baseline for the non-hGH cycles was 5.62 mm (SD 1.62) and for the hGH cycles was 4.78 (SD 1.47), ASD = 0.20. Mean endometrial thickness at trigger for the non-hGH cycles was 8.97 (SD 2.25) and for the hGH cycles was 9.27 (ASD = 0.13). 

Univariate analyses for mean endometrial growth based on varying demographic and clinical factors are shown in Table 2. Endometrial thickness gained during COH stimulation cycles had notable differences between different age and specific COH protocol subgroups, but this was not seen for ovarian reserve status or racial subgroups.

To address the primary research question, endometrial growth was compared between a total of 159 COH cycles that included hGH and 80 cycles that did not (Table 3). Patients tolerated hGH well with no report of specific side effects or adverse reactions. Mean increase in endometrial thickness was 4.5 mm (±SD 2.0) and 3.9 mm (±SD 2.3) in hGH and non-hGH groups, respectively. Therefore, in the unadjusted analysis, cycles with hGH were associated with a 0.6 mm greater increase in endometrial thickness when compared to cycles without hGH supplementation (95% CI: 0.2–1.1, *p* = 0.01). 

Demographic and cycle characteristics considered as potential confounders were subsequently controlled for in a linear mixed model linear regression, which also accounted for repeat cycles in the same patient cohort with and without hGH. After adjusting for age, BMI, DOR SART diagnosis, specific COH stimulation protocol, total gonadotropin dose, and day of trigger/peak estradiol level in the linear mixed model regression analysis, cycles with hGH were associated with a 0.9 mm greater increase in endometrial thickness when compared to cycles without hGH (95% CI: 0.4–1.4, *p* < 0.01).

## 4. Discussion

This is the first study to our knowledge to explore the impact of hGH supplementation on endometrial thickness during COH cycles with patients serving as their own controls. The adjusted regression analysis showed an almost 1 mm additional increase in endometrial thickness in COH cycles that included hGH compared to those that did not.

In assisted reproduction, hGH has most often been studied for its impact on oocyte quality as an adjunct therapy during IVF/ICSI cycles in poor-responder patients with varied results. Recent randomized controlled trials and observational studies have shown hGH has potential merit with regard to shorter stimulation course, improved oocyte yield, and greater fertilization rate/embryo conversion; however, findings regarding clinical pregnancy rates and live birth rates are not clear [21,22,23,24,25,26,27,28]. 

An additional potential benefit of hGH administration in COH cycles could be its positive effect on endometrial growth; however, the latter has not been well studied to date. hGH is a peptide hormone involved in cellular growth which is not only produced by the anterior pituitary gland for systemic endocrine effect, but also locally by reproductive tissues for local paracrine effect [16]. Endometrial expression of hGH receptors is notable in the luteal phase and theorized to correlate with progesterone levels [12]. It is postulated that hGH enhances vascularization and proliferation directly or indirectly through insulin-like growth factor 1 as well as enhances implantation through upregulated gene expression of vascular endothelial growth factor for angiogenesis and integrin beta 3 for receptivity [6,13,14,15,16]. While animal and in vitro studies have helped to elucidate these mechanisms, clinical data regarding the impact of hGH on endometrial development are lacking. Currently, such studies have been limited to case series or small retrospective studies [14,15,29]. 

The difficulty with studying hGH supplementation in assisted reproduction is in part related to inconsistencies surrounding the appropriate patient population, dosage, timing and length of administration [24,30,31,32,33]. Additionally, hGH is expensive and not FDA-approved for fertility treatment in the United States, limiting its widespread implementation and contributing to underpowered study designs [32,34,35]. Despite adequate statistical power, meta-analyses also have shown contradictory impacts on clinical outcomes, supporting the utility of additional research efforts such as ours [13,32,36,37].

As previously highlighted, a major strength of this retrospective analysis is its unique study design. By including all eligible COH cycles with hGH to comprise the treatment group and comparing them to the immediately preceding cycle without hGH to comprise the control group, patients were able to serve as their own controls, thus minimizing potential confounders. Linear mixed modeling was also implemented to further control for demographic and cycle variables that also influence endometrial thickness–age, BMI, DOR status, specific COH protocol, total gonadotropin dose, and day of trigger/peak estradiol level. In addition, all but 19 intervention cycles occurred within 1 year of the control cycle, supporting minimal time impact.

While a smaller study cohort, our data represents 5 years’ worth of IVF cycles where hGH supplementation was used. During this time, our clinical protocols have remained consistent with no major practice changes that could potentially cloud our findings. Patient are scheduled with any available provider for cycle monitoring, therefore, intra-operator differences are anticipated to be similar between the experimental and control arms. Of note, the majority of the study cohort was White or Asian, over the age of 35, and diagnosed with DOR (a commonly used indication for hGH), which limits the generalizability of our findings. Additionally, our primary outcome in the current analysis is absolute growth in endometrial thickness during a COH cycle with or without hGH, which is a surrogate marker for endometrial receptivity and pregnancy outcomes. Given the majority of embryo transfers at our institution are frozen and not fresh, IVF outcomes such as pregnancy or live birth rates were not the focus of this study since embryos were rarely transferred into the endometrial linings measured and analyzed here.

Our findings support further investigation of hGH as an adjunct therapy for patients with a history of thin endometrial lining. Intrauterine infusion of hGH has been associated with improved repair of thin endometrium in mouse models [38]. A retrospective study in patients with thin lining did not show a difference in endometrial thickness on the day of transfer in hGH vs. non-hGH cycles, but improved pregnancy rates were seen [29]. In addition, a small randomized controlled trial showed improved clinical pregnancy rates and biomarkers of endometrial receptivity following hGH administration in thin lining patients undergoing frozen embryo transfer cycles [14]. Further initiatives looking at the impact of hGH on clinical pregnancy and live birth rates during both fresh and frozen embryo transfers are imperative.

A positive impact of hGH has also been seen in recurrent implantation failure patients. Two small prospective trials focusing on specific populations of recurrent implantation failure patients have shown improved endometrial thickness and pregnancy rates-one in young patients less than 35 years of age and another in patients using donor oocytes [15,39].

The increase in use of hGH in assisted reproduction astutely raises the issue of potential risks to consider when counseling patients about introducing hGH into fertility treatments. While long-term use (years) in growth hormone deficient patients has been linked to potentially increased oncogenic and metabolic disorders, short-term, low-dose courses during assisted reproduction cycles have not had similar concerns [6]. Nevertheless, continued close monitoring is warranted. Extra caution is advised for patients with diabetes given theoretical risks of insulin resistance and poor glucose control.

In summary, hGH has the potential to positively impact not only oocyte quality, but also endometrial growth and receptivity. An approximately 1 mm improvement in endometrial thickness, as demonstrated in the hGH COH cycles included in our study, could be helpful for patients with a history of poor reproductive outcomes due to thin lining and possibly even recurrent implantation failure. An individualized approach is advised when considering the addition of hGH supplementation to embryo transfer protocols. Larger-scale, prospective trials are necessary to better comprehend the clinical impact of hGH on endometrial thickness and related pregnancy outcomes.

## Figures and Tables

**Table 1:** Study cohort demographic and clinical characteristics.

Characteristic	*n* = 80
Age at first retrieval in years, *n* (%)	
<35	8 (10)
35–37	16 (20)
38–40	23 (29)
>40	33 (41)
Body mass index (kg/m^2^), mean (SD)	23.8 (4.4)
Race, *n* (%)	
White	36 (45)
East Asian/South Asian	30 (38)/12 (15)
Black or African American	2 (2)
Ethnicity, *n* (%)	
Hispanic/Latino	7 (8.8)
Non-Hispanic/Latino	73 (91.2)
Nulliparous, *n* (%)	60 (75)
Total number of retrieval cycles, mean (SD)	3.0 (1.6)
Anti-Mullerian hormone level (ng/dL), mean (SD)	1.7 (1.7)
Total antral follicle count, mean (SD)	8.9 (4.9)
Society of Assisted Reproductive Technology Diagnosis *, *n* (%)	* Some patients had >1
Diminished ovarian reserve	55 (69)
Male factor	15 (19)
Recurrent pregnancy loss	10 (13)
Unexplained infertility	7 (9)
Tubal factor	6 (8)
Endometriosis	2 (3)
Other	2 (3)
Polycystic ovarian syndrome (poor blastocyst formation)	1 (1)
Single gene disorder	1 (1)

**Table 2:** Univariate analyses of mean increase in endometrial thickness.

Characteristic	*n* (239 Total Cycles)	Endometrial Growth in mm, Mean (SD)	ASD *
Age at retrieval in years			0.47
<35	15	5.5 (2.3) baseline 5.0 (1.6), trigger 10.5 (1.8)	
35–37	38	3.9 (2.8) baseline 4.8 (1.8), trigger 8.7 (2.7)	
38–40	82	3.7 (1.7) baseline 4.5 (1.3), trigger 8.1 (2.0)	
>40	104	4.8 (2.0) baseline 5.2 (1.5), trigger 10.0 (2.2)	
Race			0.22
White	89	4.0 (2.0)	
East Asian/South Asian	88/56	4.7 (2.4)/4.0 (1.8)	
Black or African American	6	4.7 (1.8)	
Ovarian Reserve			0.41
Diminished ovarian reserve	177	5.0 (2.4)	
Non-diminished ovarian reserve	62	4.1 (2.0)	
Controlled ovarian hyperstimulation protocol			0.60
Antagonist	119	4.2 (2.2)	
Microdose flare	76	4.3 (2.2)	
Clomid antagonist	22	3.9 (1.8)	
Letrozole antagonist	18	4.9 (2.1)	
Long Lupron	4	6.4 (1.5)	

* ASD = Absolute standardized difference; this represents the difference in means between the groups divided by the pooled standard deviation. The values 0.2, 0.5, and 0.8 correspond to small, medium, and large differences, respectively. Therefore, a smaller ASD signifies less difference between the groups.

**Table 3:** Unadjusted and adjusted linear mixed models-endometrial growth in human growth hormone (hGH) cycles is reported (as compared to non-hGH cycles).

Model	Endometrial Growth in mm, Estimate	Lower 95% CI	Upper 95% CI	*p*-Value
hGH (unadjusted)	+0.6	+0.2	+1.1	0.01
hGH (adjusted) *	+0.9	+0.4	+1.4	<0.01

* Controlled for age at retrieval, body mass index, diminished ovarian reserve status, controlled ovarian hyperstimulation protocol, total gonadotropin dose, and day of trigger/peak serum estradiol level.

## Data Availability

The data presented in this study are available on request from the corresponding author.
